# Transmembrane Protease Serine 2 Proteolytic Cleavage of the SARS-CoV-2 Spike Protein: A Mechanistic Quantum Mechanics/Molecular Mechanics Study to Inspire the Design of New Drugs To Fight the COVID-19 Pandemic

**DOI:** 10.1021/acs.jcim.1c01561

**Published:** 2022-05-12

**Authors:** Luís M. C. Teixeira, João T. S. Coimbra, Maria João Ramos, Pedro Alexandrino Fernandes

**Affiliations:** LAQV/REQUIMTE, Departamento de Química e Bioquímica, Faculdade de Ciências Universidade do Porto, Rua do Campo Alegre, s/n, 4169-007 Porto, Portugal

## Abstract

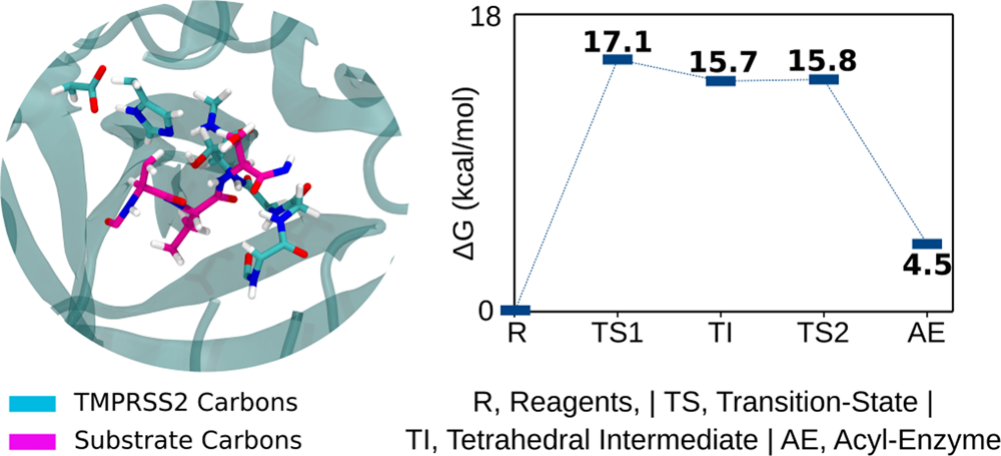

Despite the development of vaccines against the severe acute respiratory syndrome coronavirus 2 (SARS-CoV-2) virus, there is an urgent need for efficient drugs to treat infected patients. An attractive drug target is the human transmembrane protease serine 2 (TMPRSS2) because of its vital role in the viral infection mechanism of SARS-CoV-2 by activation of the virus spike protein (S protein). Having in mind that the information derived from quantum mechanics/molecular mechanics (QM/MM) studies could be an important tool in the design of transition-state (TS) analogue inhibitors, we resorted to adiabatic QM/MM calculations to determine the mechanism of the first step (acylation) of proteolytic cleavage of the S protein with atomistic details. Acylation occurred in two stages: (i) proton transfer from Ser441 to His296 concerted with the nucleophilic attack of Ser441 to the substrate’s P1-Arg and (ii) proton transfer from His296 to the P1′-Ser residue concerted with the cleavage of the ArgP1-SerP1′ peptide bond, with a Gibbs activation energy of 17.1 and 15.8 kcal mol^–1^, relative to the reactant. An oxyanion hole composed of two hydrogen bonds stabilized the rate-limiting TS by 8 kcal mol^–1^. An analysis of the TMPRSS2 interactions with the high-energy, short-lived tetrahedral intermediate highlighted the limitations of current clinical inhibitors and pointed out specific ways to develop higher-affinity TS analogue inhibitors. The results support the development of more efficient drugs against SARS-CoV-2 using a human target, free from resistance development.

## Introduction

Since the World Health Organization, in January 2020, declared the severe acute respiratory syndrome coronavirus 2 (SARS-CoV-2) as a public health emergency of international concern,^1^ several efforts have been made to improve the diagnostic and treatment of this viral infection. However, even though vaccination plans are now used all over the world, several factors, such as the slow pace of vaccination, the fraction of the population that does not develop immunity upon vaccination, and the very good but not total efficiency of vaccines in preventing infection, result in a high number of daily cases, for which effective drug treatment is still lacking.^2^ As a result, even some infected vaccinated patients still have an associated risk of significant morbidity and even mortality related to this viral infection.^3^ It is thus urgent to understand the biological mechanisms underlying this disease and to discover alternative or novel therapeutic targets.

SARS-CoV-2 is an enveloped virus with a single-stranded positive-sense RNA genome that contains four key structural proteins, spike (S protein), envelope (E protein), membrane (M protein), and nucleocapsid (N protein), and 16 nonstructural proteins.^4^ The virus surface is covered by glycosylated S proteins, which bind to the host cell receptor angiotensin-converting enzyme 2, mediating viral entry.^5^

The S protein of SARS-CoV-2 is composed of two domains: (i) S1, which is the receptor-binding domain, and (ii) S2, which contains functional elements required for the membrane fusion. It has two cleavage sites, one at the S1/S2 border and another in the S2 domain. The S1/S2 border cleavage site is composed of multiple Arg residues, and it is in an exposed multibasic loop.^6^ The S protein requires a conformational change upon binding the host cell receptor to allow the fusion between virus and the host cell membrane. This conformational change is promoted through proteolytic cleavage by human proteases, such as the human transmembrane protease serine 2 (TMPRSS2). Because TMPRSS2 is anchored to the plasma membrane, it is the principal protease promoting viral fusion through proteolytic cleavage of the S protein. Although this is the primary mechanism of cell entry used by SARS-CoV-2, if TMPRSS2 is not present on the host cell surface, the virus uses a slower and less efficient viral entry process based on endosomal proteases to trigger endocytosis.^7^

Furthermore, this serine protease participates in several physiological functions such as tissue remodeling, blood coagulation, inflammatory responses, digestion, and fertility.^8^ In fact, TMPRSS2 has been associated with several other pathological mechanisms.^9^ For that reason, although this protease has physiological functions, inhibitors have been developed to regulate its activity in some pathological contexts. For example, in the case of androgen-responsive prostate cancer, the inhibition of TMPRSS2 was beneficial in stopping tumor metastasis.^10^

Because viral protein targets generally have high mutation rates and quickly develop resistance through mutation and natural selection, having a human enzyme (such as TMPRSS2) as a therapeutic target, free from these mechanisms of resistance development, poses obvious advantages over viral-target alternatives.

It has been observed that the TMPRSS2 inhibitors camostat and nafamostat (both inhibitors bind covalently to TMPRSS2^11^) effectively block the SARS-CoV-2 viral entry in cell lines.^12^ Furthermore, nafamostat not only exhibited a higher potency than camostat, but it also has proven capable of inhibiting SARS-CoV-2 infection in two COVID-19 mouse models, leading to a better disease outcome in these cases.^13^ These results suggest that TMPRSS2 is a possible therapeutic target in treating SARS-CoV-2 infection.^14^ Recently, phase-II clinical trials for COVID-19 treatment with nafamostat have been conducted. The study failed to find a significant difference in time to clinical improvement. However, a shorter median time to clinical improvement in a small group of high-risk patients was reported. Thus, with the purpose of assessing the efficacy of the treatment with nafamostat, a phase-III clinical trial has been warranted.^15^ Having this in mind, it may be helpful to discover alternative compounds that behave as competitive inhibitors, not only to enrich the pool of possibilities for SARS-CoV-2 infection treatment, but also due to the more significant side effects that irreversible inhibitors usually have. It would be useful also to design covalent inhibitors with improved affinity, as higher affinity translates into higher specificity, something fundamental to reduce the side effects of covalent inhibitors.

TMPRSS2 has 492 amino acid residues and is composed of a type-2 transmembrane domain, a low-density lipoprotein receptor class A domain that binds calcium, a scavenger receptor cysteine-rich domain involved in the binding to other cell surfaces or extracellular molecules, and a serine protease domain from the S1 family that cleaves Arg or Lys residues.^16^ The N-terminal region is located in the cell cytoplasm, while the serine protease domain is in the extracellular region.^8^ Although the catalytic domain is linked to the membrane-bound portion of the enzyme through a disulfide bond, when TMPRSS2 is activated, this domain is released into the extracellular space. The catalytic domain is responsible for the cleavage of cell-membrane receptors, cytokines, growth factors, and extracellular matrix components.^17^

**Scheme 1:**
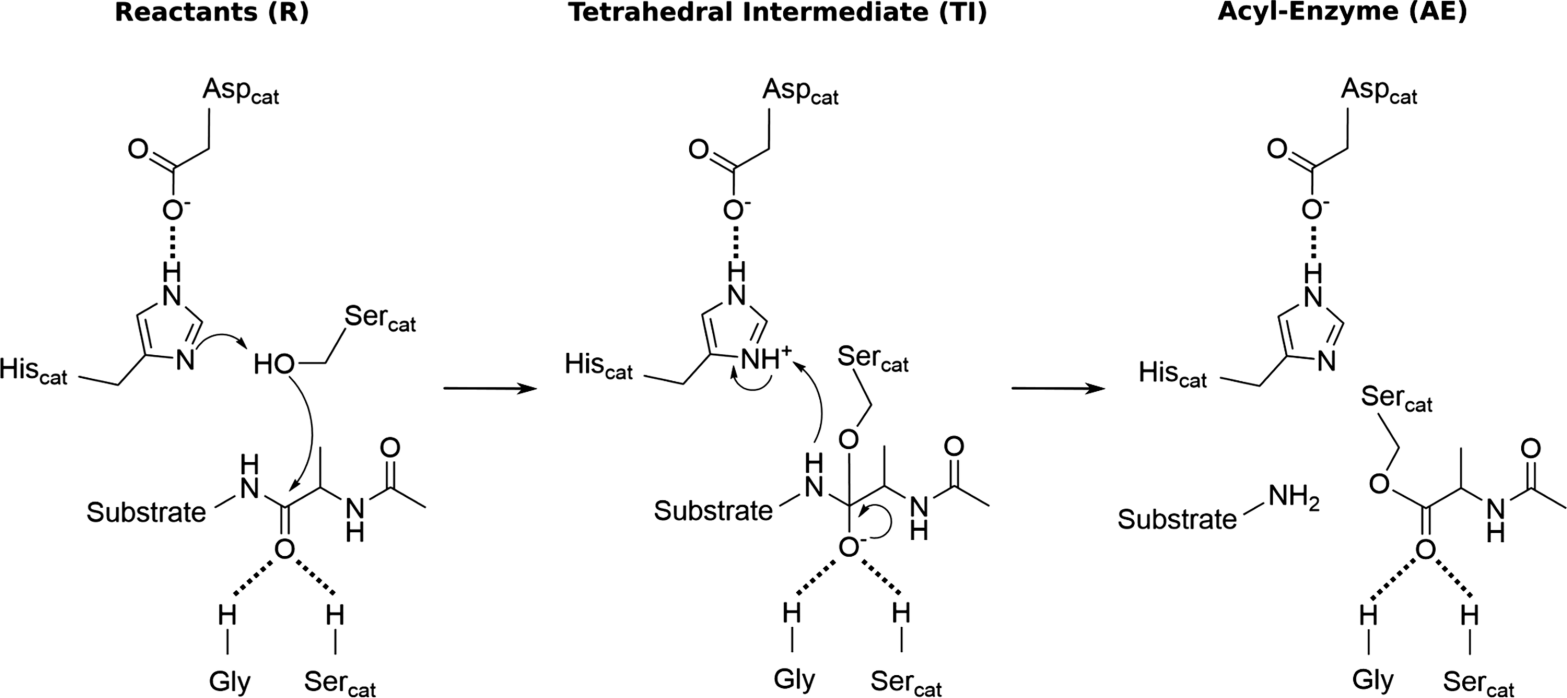
General Acylation Step for Trypsin-like Proteases^*a*^ This step involves a proton transfer and a nucleophilic attack, leading to the formation of a TI. The oxyanion hole residues (a nearby Gly and the backbone of Ser_cat_) stabilize the newly formed TI through hydrogen bond interaction. Afterward, a peptide bond is cleaved, leading to the formation of an AE.

The general reaction mechanism of trypsin-like and chymotrypsin-like serine proteases has been extensively studied, theoretically and experimentally.^18^ The mechanism usually is composed of two steps: (1) an acylation step and (2) a deacylation step. The acylation step (Scheme 1) starts with deprotonation by the His_cat_ imidazole ring of the Ser_cat_ nucleophilic hydroxyl group. Meanwhile, the Asp_cat_ side-chain stabilizes the positively charged His_cat_. A nucleophilic addition on the substrate carbonyl carbon by Ser_cat_ hydroxyl group oxygen leads to a tetrahedral intermediate (TI). The proton transfer may or not be concerted with the nucleophilic addition. The newly formed TI state is stabilized through hydrogen bonding to the backbone of nearby residues that form an oxyanion hole. Subsequently, the peptide bond is broken, liberating the substrate’s N-terminal, and the acyl-enzyme (AE) is formed. The deacylation step leads to the formation and the release of the product and regeneration of the enzyme catalytic residues.^19^ For this reaction mechanism, the acylation step was proposed to be the rate-limiting step.^20^

Even though the general mechanism of serine proteases is well studied, its atomistic details are not the same for every family member. However, such atomic-level details are needed for the rational design of high-affinity transition-state analogue inhibitors. To the best of our knowledge, the enzymatic reaction catalyzed by TMPRSS2 for the cleavage of the SARS-CoV-2 S protein has not yet been described with such atomistic details.

The objective of this work was to study the mechanistic reaction of the rate-limiting acylation step of the reaction catalyzed by TMPRSS2, identify the rate-limiting states of the mechanism, and derive specific indications about how to develop transition-state (TS) analogue inhibitors based on the rate-limiting TSs. For this purpose, an adiabatic quantum mechanics/molecular mechanics methodology (QM/MM) was employed. This approach has several advantages and limitations when compared with closely related methods that are often used to describe enzymatic reactions, such as QM/MM molecular dynamics (MD).^21−24^ Adiabatic QM/MM methods account for the effects of the explicit enzymatic environment and can employ a high or very-high-level Hamiltonian in a significantly large QM region but, on the other side, are more limited in the sampling of the very complex enzymatic conformational space. This can be accounted for, to some extent, by employing different initial conformations (often extracted from classical MD simulations), in the so-called multiple potential energy surface (multi-PES) QM/MM approach.^22,25−27^ Still, adiabatic QM/MM methods, either single-conformation or multiconformation, continue to provide excellent results in the study of enzymatic reactions.^21^ Moreover, a recent study has shown that the multi-PES QM/MM and QM/MM MD lead to equivalent results if the QM layer is represented at the density functional theory (DFT) level in both.^28^

We have characterized all stationary points for the first step of the reaction catalyzed by TMPRSS2 and provided detailed atomistic and thermodynamic results. The results were in line with other experimental and theoretical studies on serine proteases. Subsequently, we have identified the enzyme’s key TI state interactions that ought to be included in high-affinity, TS analogue inhibitors of TMPRSS2. We show that these interactions are unexplored on the current clinical inhibitor for which structural information is available. The latter mimic the lower-affinity substrate but not the high-affinity TS. Thus, the results obtained in this work will help in the development of drugs for the treatment of COVID-19.

## Experimental Methods

### System Preparation

The coordinates of the serine protease TMPRSS2 were retrieved from its X-ray structure deposited in Protein Data Bank (PDB ID: 7MEQ).^29^ We kept only the catalytic domain in the mechanistic study (residues 256–491). To study the TMPRSS2 enzymatic reaction mechanism, we modeled an enzyme:substrate complex using an octapeptide with the sequence PSKRSFIE. The octapeptide corresponded to the P4-P3-P2-P1-P1′-P2′-P3′-P4′ positions of the SARS-CoV-2 S protein S1/S2 border cleavage site. It was retrieved from the work by Huggins.^30^ The modeling was performed using Open-source PyMOL (version 2.3.0), where the C^α^s from the crystallographic structure and the retrieved structure were aligned. Then, the coordinates of the octapeptide were transferred to the X-ray structure. Finally, the two water molecules from the crystallographic structure numbered 728 and 806 were removed to avoid clashes with the modeled octapeptide. This modeling strategy was validated by the alignment between the X-ray structure and the homology structure from Huggins’ work. The superimposed structures revealed a shared resemblance, especially in the active site region (Figure S1).

Afterward, a prediction of the p*K*_a_ of titratable residues was performed using the DelPhiPKa web server.^31^ Based on the p*K*_a_ predictions, the reaction mechanism, and the chemical environment, His274, His296, and His307 were set as the neutral form with a protonated Nδ. In contrast, His279 and His334 were protonated at the Nε. In addition, Asp458 was also protonated to its neutral form. The ff14SB AMBER force field^32^ was employed to generate the bonded and nonbonded parameters of the protein and the peptide substrate. The system was solvated in an isometric box of 16,000 TIP3P water molecules^33^ and was neutralized with three chloride counter ions.

### System Minimization

In this work, before the QM/MM calculations, the system was minimized and relaxed in three steps: (i) minimization of water molecules, (ii) water relaxation, and (iii) minimization of the entire system. For the water molecules minimization and water relaxation steps, the rest of the atoms in the system were restrained, using a harmonic restrain force constant of 5.0 kcal/mol Å^2^. The water minimization consisted of 20,000 cycles, in which the first half employed the steepest descent algorithm and the other half the conjugated gradient algorithm. As for the water relaxation stage, we have simulated the system for 20 ps. The initial temperature of the system was set to 200.0 K, which was gradually increased to 300.0 K during this stage. The temperature was maintained with a weak-coupling algorithm. The system was also maintained at a constant pressure using isotropic position scaling with a pressure relaxation time of 1.0 ps and controlled by a Berendsen barostat.^34^ The bonds involving hydrogen atoms were constrained with the SHAKE algorithm.^35^ The system minimization was identical to the first minimization step, but no restraints were employed in this case, other than the SHAKE algorithm constraint for the bonds involving hydrogen atoms. For all three steps, a nonbonded cutoff of 10 Å was employed.

### QM/MM Calculations for the First Acylation Step

The QM/MM calculations model was built with the VMD^36^ plugin molUP,^37^ using the previously obtained minimized structure. This structure was validated through a 100 ns MD simulation, which led to the conclusion that the geometry of the system, and in particular, the reaction-participating atoms were stable. In fact, the average RMSd of the protein backbone atoms was 1.60 Å, and the average RMSd of the QM region atoms was 0.65 Å (SI—Molecular Dynamics Simulation Analysis: Figures S2–S6).

The QM/MM model was composed of the TMPRSS2 catalytic domain, the octapeptide substrate, and all water molecules within a 3.0 Å radius of any atom of the enzyme:substrate complex (740 water molecules). The Our own N-layered Integrated Molecular Orbital/Molecular Mechanics (ONIOM) methodology was employed in every calculation.^38^ The model system was divided into the high-level (HL) and the low-level (LL) layers. The HL layer was described at the DFT level, while the LL layer was described with MM.

**Figure 1:**
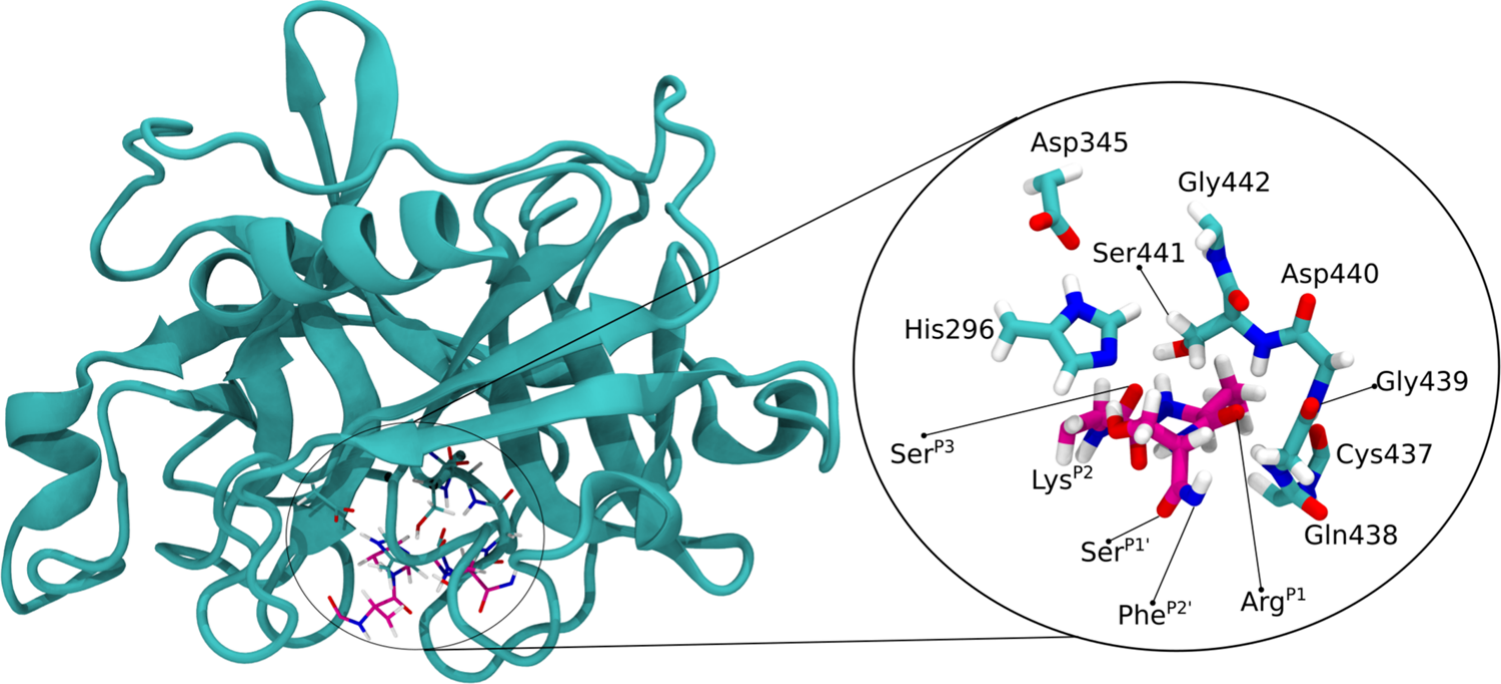
Representation of the ONIOM model and of the HL layer atoms. Enzyme carbon atoms are shown in cyan and octapeptide carbon atoms in magenta. The water molecules were omitted for clarity.

The HL layer consisted of 90 atoms, including atoms from the catalytic triad, His296, Asp345, and Ser441, a few neighboring residues, Gly442, Asp440, Gly439, Gln438, and Cys437, and atoms from the substrate residues of the positions P3, P2, P1, P1′, and P2′ (Figure 1). Hydrogen atoms were used as link atoms to complete the valence of the bonds located in the border between the two layers. Except for three water molecules that were very close to the HL layer, all water molecules were frozen.

The model was submitted to two geometry optimization steps. The first optimization was made with the mechanical embedding scheme, and the second optimization, starting from the previously optimized structure, was made with the electrostatic embedding scheme. Subsequently, the last optimized structure was retrieved and subjected to a potential energy surface scan (the reaction was explored through the system’s evolution as a function of a reactional coordinate implicated in each reactional step). The DFT functional used in these calculations was B3LYP^39^ with the basis set 6-31G(d), both available in Gaussian 09.^40^ The quadratic coupling scheme was employed in these calculations.

The chosen reaction coordinate for the first reactional step was the nucleophilic attack from the Ser441 oxygen to the substrate’s carbonyl carbon of Arg^P1^. The interatomic distance was decreased from 2.76 to 1.41 Å, with steps of −0.05 Å. After the scan, the TS, the structure with the highest energy obtained in the scan, was further optimized without restraints to obtain a reaction coordinate-free TS. The TS was confirmed by vibrational frequency calculations, resulting in a single imaginary frequency. Then, both the reactant (R) and the TI were determined using an internal reaction coordinate (IRC) procedure^41^ and were further optimized. All relative energies were retrieved from the optimized structures of the R, TS, and TI. The zero-point energy, entropy, and thermal energy were calculated within the rigid rotor/harmonic oscillator approximation to retrieve the Gibbs free energy of the system.

A multiple conformation QM/MM study was also conducted to evaluate the dependence of the starting structure conformation on the first reactional step. In this regard, 19 distinct conformations were retrieved from a 100 ns MD simulation. The sum of the interatomic distances *d*_sum_ = *d*(His296 Nε – H1 Ser441) + *d*(Ser441 Oγ – C1 Arg^P1^) was used as the criterium to select these conformations. The values of *d*_sum_ for the 19 structures were within [4.22, 5.42] Å, whereas for the minimized structure *d*_sum_ was 4.82 Å. Interestingly, 75% of the structures obtained from the MD simulation met this criterion. Each structure was then subjected to a potential energy surface scan. These calculations were carried out at the B3LYP/6-31G(d):FF14SB level of theory. The reaction coordinate was identical to the one chosen in the minimized structure (the nucleophilic attack from the Ser441 oxygen to the substrate’s carbonyl carbon of Arg^P1^). The energy difference was calculated through single-point energy calculations (using M06-2X/6–311++G(2d,2p):FF14SB), to determine the value of the energetic barrier associated with that specific conformation. The reported values in this case only refer to the internal energies retrieved from the minima and maxima obtained from the linear transit scan. The contribution of the rigid rotor/harmonic oscillator corrections should have a small contribution to the activation free energy.^26^

To continue the acylation reaction mechanism, we applied the same protocol as for the first stage of this step. For the second scan, the reaction coordinate was defined as the peptide bond cleavage of the substrate. The interatomic distance was increased from 1.50 to 2.90 Å, with steps of 0.05 Å. However, the TI obtained through the second IRC calculation showed an ONIOM energy difference of 1.9 kcal mol^–1^ in relation to the one obtained from the decay of the first TS. The main difference between the two structures was a slight change in the conformation of His296 in the function of the substrate’s Arg N1 (Figure S7A). We have then performed a reverse scan of the first reactional step, starting from the TI structure obtained by the second IRC. After repeating the methodology mentioned above, the newly TI optimized structure was compared with the TI structure obtained from the first IRC calculation. This time the ONIOM energy difference lowered to 1.4 kcal mol^–1^, but the conformation of His296 was similar (Figure S7B). Thus, the R, TS, and TI structures considered were the latter obtained, that is, the ones derived from the methodology applied to the reverse scan.

Single-point energy calculations were performed in the five stationary points: (i) R, (ii) TS1, (iii) TI, (iv) TS2, and (v) AE. The ONIOM, QM, and MM energies of the acylation step are shown in the Supporting Information (Figure S8 and Table S1). For these calculations, we used the M06-2X/6–311++G(2d,2p):FF14SB//B3LYP/6-31G(d):FF14SB level of theory. The M06-2X functional was chosen because of two reasons: (i) it has been shown that M06 functionals perform better than B3LYP for systems with dispersion and hydrogen-bonding interactions^42^ and (ii) M06-2X has been proven to be one of the most accurate density functionals for proton transfer reactions between different amino acids.^43^ The atomic charges for the stationary points were obtained using a Hirshfeld population analysis.^44^ We also performed single-point energy calculation for each of the five stationary points adding the D3 dispersion. However, we observed that the energies obtained by M06-2X/6–311++G(2d,2p):FF14SB//B3LYP/6-31G(d):FF14SB with or without the D3 dispersion were similar (see Table S2).

An energy reassessment study was performed to the R and TS1 state to evaluate the contribution of the oxyanion hole to the stabilization of the TS1 state. The oxyanion hole hydrogen bond interactions were “deleted,” by replacing both -NH groups of Ser441 and Gly439 with -CH_2_ groups. This substitution allowed us to evaluate the impact of the oxyanion hole hydrogen bond interactions on the Gibbs activation barrier. Subsequently, we optimized the newly obtained structures with the electrostatic embedding scheme. This optimization was performed with all atoms frozen except for the two added -CH_2_ groups. Afterward, single-point energy calculations were performed at the M06-2X/6–311++G(2d,2p):FF14SB//B3LYP/6-31G(d):FF14SB level of theory.

## Results and Discussion

According to our calculations, the acylation step catalyzed by TMPRSS2 occurred in two sequential stages that will be further detailed. All energies will be discussed at the M06-2X/6–311++G(2d,2p):FF14SB//B3LYP/6-31G(d):FF14SB level of theory. Our optimized QM/MM model starting from a TMPRSS2:octapeptide modeled complex showed a correct alignment of the active site residues for the proteolytic reaction to begin with, that is, (i) the catalytic His296 and Ser441 were interacting, and (ii) Ser441 was oriented to Arg^P1^, where the proteolytic cleavage occurs.

### First Stage of the Acylation Step

**Figure 2:**
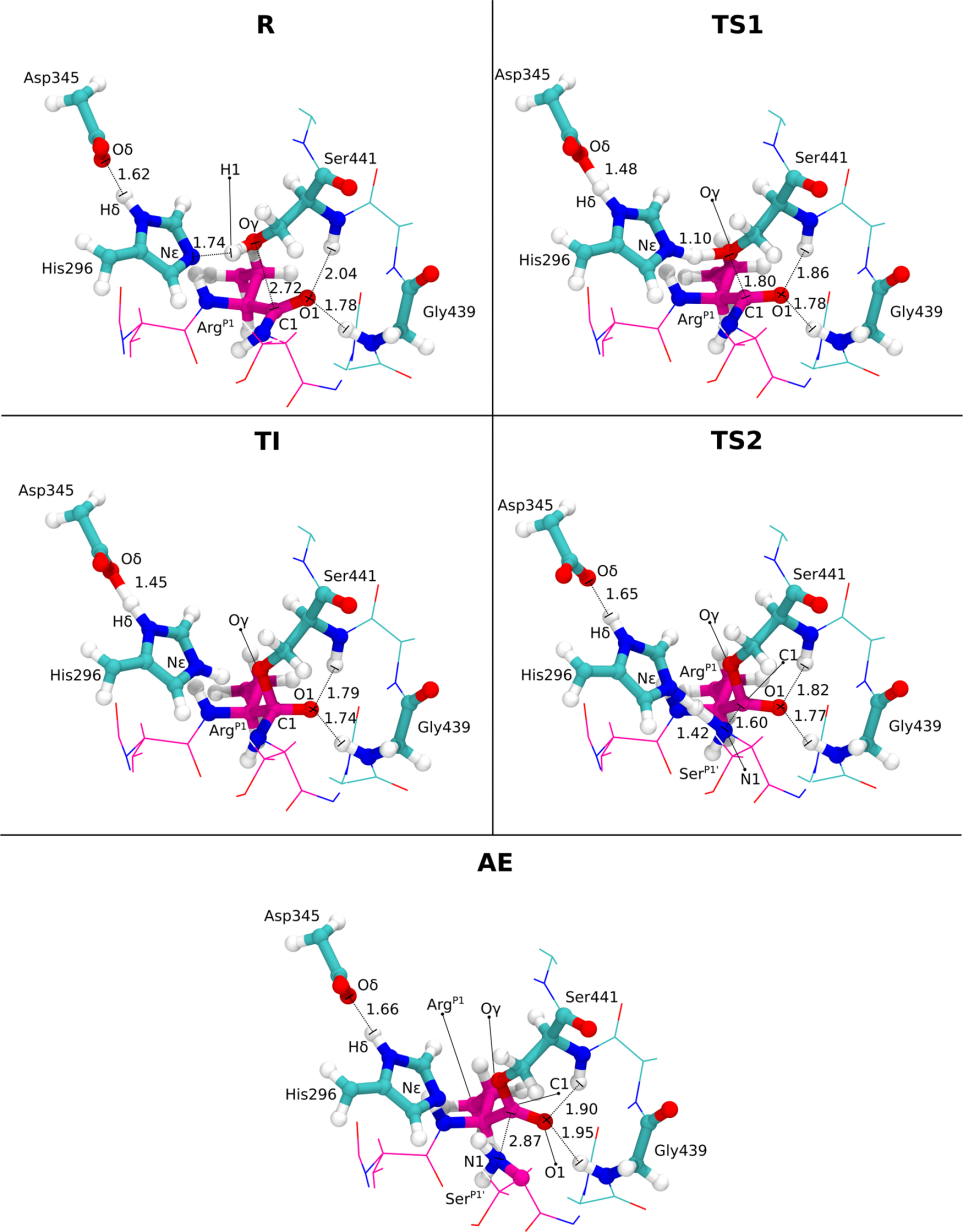
Acylation step stationary points. The stationary points of the two stages of the acylation step: (i) proton transfer between His296 and Ser441, and nucleophilic attack of Ser441 to Arg^P1^; (ii) proton transfer between His296 and substrate’s Ser^P1′^, and cleavage of the Ser^P1′^-Arg^P1^ peptide bond. The protein’s carbon atoms are represented in cyan, and the substrate’s carbon atoms are represented in magenta. Critical reaction distances are represented in Å.

In the first stage of the acylation step, the proton of the hydroxyl group of Ser441 was transferred to His296 in a concerted manner with the nucleophilic attack of Ser441 to the carbonyl carbon of Arg^P1^. This was characterized by an activation Gibbs energy barrier (Δ*G*^‡^) of 17.1 kcal mol^–1^. The first transition state (TS1) presented an imaginary frequency of 293*i* cm^–1^. This first stage led to the TI, which was highly endergonic, at 15.7 kcal mol^–1^ relative to the R. During this step, and by following the atomic charges of the main atoms involved in the reaction, the atomic charge on the carbonyl oxygen of Arg^P1^ (O1) went from −0.26 a.u at the R state, to −0.34 a.u at the TI state (Figure S9). This favored the stabilization by the oxyanion hole residues, Ser441 and Gly439, as it can be seen by the reduction of the hydrogen bond distances to O1, changing from 2.04 to 1.79 Å and from 1.78 to 1.74 Å, respectively (Figure 2). The atomic charge of His296 Nε went from −0.20 to −0.06 a.u, meaning that during the first stage of the acylation step, there was an increase in the cationic nature of this atom and consequently of the acidity of His296. This residue was also further stabilized by Asp345. In fact, the distance between the closest oxygen of the carboxylic group of Asp345 and the Hδ of His296 decreased from 1.62 to 1.45 Å.

Throughout the first stage, the atomic charge of Ser441’s Oγ went from −0.24 to −0.18 a.u, while the atomic charge of Arg^P1^ carbonyl carbon (C1) went from +0.20 to +0.18 a.u. As we can see, C1 tended to become slightly more negative throughout the first stage, while Oγ tended to become more positive. This meant that the covalent bond between these two atoms, required for the formation of TI, was being formed.

We observed that the variations in the atomic charge for the six atoms analyzed throughout the first stage were in accordance with the expected tendencies for the first-stage reactions (proton transfer and nucleophilic attack). However, the absolute numbers of these variations were not the ones expected, so we decided to evaluate the charge variation of all HL atoms of His296, Ser441, the peptide, and Asp345. The variation of the His296 charge throughout this first stage was +0.5 a.u.; the variation of Ser441 was −0.7 a.u.; for the peptide the variation was −0.6 a.u. Finally, the charge variation of Asp345 was +0.1, which meant that this residue did not suffer a significant charge variation throughout this stage. Overall, the charge variations correspond to what is expected based on chemical intuition for the mechanism shown in Scheme 1. These results (shown in Figure S10) indicated that the electron donation led to a decrease in charge of Ser441, which favored the nucleophilic attack. This nucleophilic attack also led to a decrease in the charge of the peptide because of the formation of a covalent bond between Ser441 Oγ and the substrate’s Arg^P1^. The increasing positive charge of His296 is stabilized by the neighboring Asp345 carboxylate group (evidenced by the decrease of the interatomic distance throughout the first stage).

The first stage results that led to the formation of the TI were in line with both experimental and computational results in the literature for this type of system. Experimentally, the Gibbs activation energy for serine proteases varies from 15 to 20 kcal mol^–1^, depending on the reaction conditions (for example, such as temperature and pH) and on the substrate.^45^

In the work of Nutho et al.^46^ on the Zika virus NS2B/NS3 serine protease, a concerted first step was observed, which led to the formation of the TI, with a barrier of 16.3 kcal mol^–1^. Stabilization of the substrate’s Arg^P1^ carbonyl oxygen by an oxyanion hole and a stabilization of His_cat_ by Asp_cat_ was also observed. Concerning the atomic charges, the tendencies of His296 Nε, Arg^P1^ O1, and SerP1′ N1 were similar. However, the tendencies of Ser441 Oγ and H1 and Arg^P1^ C1 were different. This difference is probably explained by the different methodology and levels of theory employed in both studies (Nutho et al.^46^ analyzed the average Mulliken charges of QM/MM MD umbrella sampling simulations at the PM6/ff14SB level of theory).

In the work of Lima et al.^47^ on the Dengue virus NS2B/NS3pro serine protease, a higher Gibbs activation barrier for the formation of the TI – 33 kcal mol^–1^ was observed. The energy difference between this result and the one shown here could be related to the methodology and level of theory employed. Lima et al.^47^ employed a QM/MM MD and umbrella sampling methodology and treated the HL with a semiempirical Hamiltonian (PDDG/PM3). In contrast to our results, the first step occurred in a nonconcerted manner, with a first stage leading to the deprotonation of Ser_cat_ (with a barrier of 24.1 kcal mol^–1^), and a second stage, proceeding to the formation of the TI (with a barrier of 10.9 kcal mol^–1^). A proton transfer between His_cat_ and Asp_cat_, was also observed, which was proposed to increase the nucleophilicity of His_cat_ and favor the activation of Ser_cat_. Concerning the atomic charges of the main atoms involved in the reaction, both studies agreed about the tendencies of His296 N^ε^, Arg^P1^ O^γ^, and SerP1′ N1 charges. Despite the tendency of Ser441 O^γ^ from the R to the TS not being in accordance, the atomic charge of this atom was more positive in the TI state (last state of the first stage) compared with the R state (first state). This meant that the tendency on the TI state was in line with the one obtained in our work.

### Second Stage of the Acylation Step

In the second stage of the acylation step, the proton from Ser441 was transferred to Ser^P1′^ in a concerted manner with the cleavage of the ArgP1-SerP1′ peptide bond. In this case, the Gibbs energetic barrier was 15.8 kcal mol^–1^, relative to the R state. The second transition state (TS2) was also further characterized and presented a single imaginary frequency of 1022*i* cm^–1^. The second stage led to the formation of the AE, which was less endergonic than the TI stationary point; that is, it was 4.5 kcal mol^–1^ more energetic than R (and −11.3 kcal mol^–1^ more stable than the TI). Throughout this stage, the atomic charge of Arg^P1^ O1 went from −0.34 to −0.24 a.u. This atomic charge increase led to the increase of the interatomic distance of the oxyanion hole interactions: (i) The Arg^P1^-Ser441 distance increased from 1.79 to 1.90 Å, and (ii) the Arg^P1^-Gly339 distance increased from 1.74 to 1.95 Å. Throughout the second stage, His296 Nε atomic charge went from −0.06 to −0.22 a.u, which led to the decrease of this amino acid’s cationic nature and acidity. Consequently, the distance of Asp345 carboxyl carbon to His296 H^δ^ increased from 1.45 to 1.66 Å.

Throughout the second stage, the atomic charge of Arg^P1^ C1 went from 0.18 to 0.25 a.u. Meanwhile, SerP1′ N1 atomic charge went from −0.21 to −0.23 a.u. These inversed atomic charge tendencies accompanied the breaking of the ArgP1-SerP1′ peptide bond. The decrease of the atomic charge of SerP1′ N1 also favored the proton transfer.

Throughout the second stage of the acylation step, the atomic charge tendencies for the key involved atoms agreed with the expected charge variation for this stage. However, just as the first stage, the absolute numbers of these variations were not the ones expected. Once again, we evaluated the charge variation for His296, Asp345, and the peptide. We observed a variation of −0.5 a.u for His296, a variation of −0.1 a.u for Asp345, and +0.7 for the peptide. In comparison with the ones obtained for the first stage, these results showed an inversed variation. An electron was accepted by His296, which led to a decrease in its atomic charge. This decrease was compensated by weakening the interaction strength between His296 Hδ and Asp345 carboxyl oxygen (evidenced by an increase of the interatomic distance). The electron donation and the peptide bond cleavage between the substrate’s Arg^P1^-Ser^P1′^ led to an increase of the atomic charge of the peptide (Figure S10).

Similar to what was observed in the first acylation stage, the results obtained in this work were in accordance with those in the literature. In the work of Nutho et al.,^46^ a difference between the TI and TS2 of 2.0 kcal mol^–1^ was observed. It was also observed that the proton transfer and the peptide bond cleavage happened simultaneously, in line with the findings of our work.

In the work of Lima et al.,^47^ a difference between TI and TS2 of 5.1 kcal mol^–1^ was observed. Contrary to our study, there was a rearrangement between Asp_cat_ and a nearby His residue in their work, leading to a barrier and minimum between TI and TS2. A proton transfer between His_cat_ and the substrate happened in a concerted manner with the cleavage of the substrate’s peptide bond, in line with our results.

After the first stage, the enzyme-intermediate covalent complex undergoes hydrolysis to generate the final product.^19^ Because in trypsin-like serine proteases, the rate-limiting step of the proteolytic cleavage mechanism is the acylation step,^20^ our objective was restricted to obtaining detailed atomistic information about the TS of the rate-limiting step. The detailed information on TS1 may then support the development of alternative therapeutic routes for the treatment of COVID-19. Thus, the deacylation step is beyond the objectives of the work and thus was not studied here.

**Figure 3:**
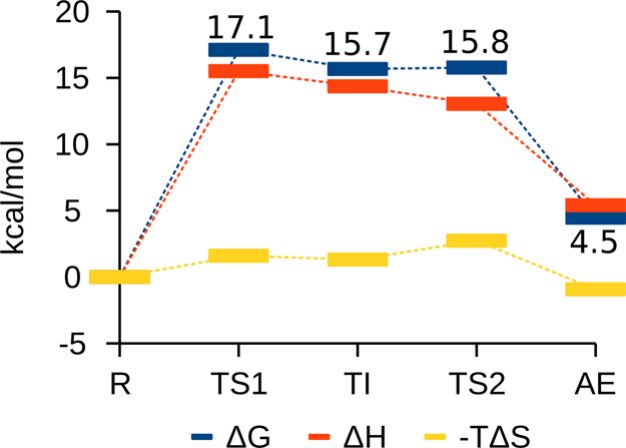
Thermochemical profile for the acylation step by TMPRSS2. The Gibbs energy for each stationary point is highlighted.

Figure 3 shows that the acylation step was overall an endothermic process (AE state had an energy of 4.5 kcal mol^–1^ higher than R) with an activation Gibbs energy barrier of 17.1 kcal mol^–1^ corresponding to the TS1 state (formation of the TI). Furthermore, the thermochemical profile showed that the enthalpy had the highest contribution to the reaction. The highest enthalpic state was TS1, where the bond between Ser441 Oγ and Arg^P1^ was being formed, and the highest entropic state was TS2, where the ArgP1-SerP1′ peptide bond was being broken. The difference between TI and TS2 was 0.1 kcal mol^–1^, smaller than RT, suggesting an extremely short lifetime for TI, and an almost barrierless transition through TS2 to AE at physiological temperature. Experimentally, it is known that the TI of serine proteases is difficult to obtain due to its formation being energetically unfavorable, so a very quick transition between TI and TS2 is in agreement with the experimental data.^48^

To understand the influence of the starting reactant conformation on the rate-limiting step of the reaction catalyzed by TMPRSS2, we have performed a multi-PES QM/MM analysis. In this regard, we selected 19 structures from a 100 ns MD simulation that covered well the simulation time scale, spanning almost the entire simulation of 100 ns. These were selected based on a simple criterion, *d*_sum_, which was defined by the sum of the proton transfer and nucleophilic attack distances that govern the first reactional step, *d*_sum_ = *d*(His296 Nε – H1 Ser441) + *d*(Ser441 Oγ – C1 Arg^P1^).

**Figure 4:**
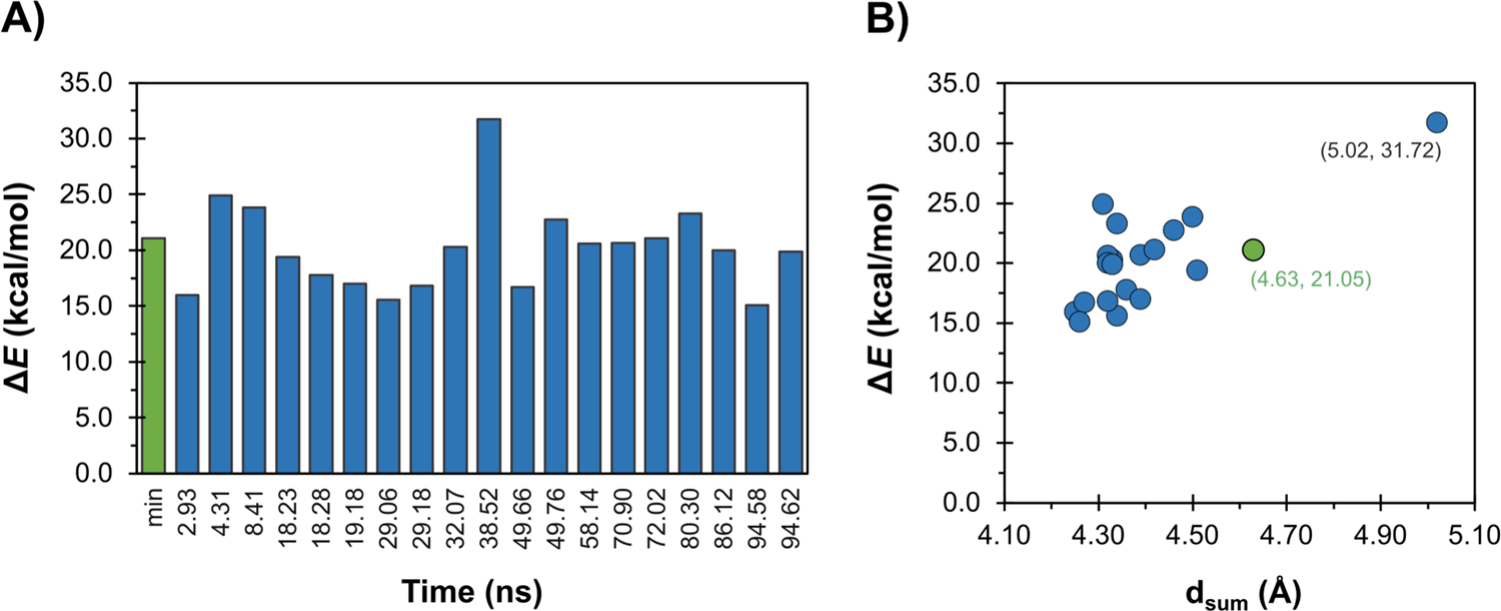
multi-PES results. (A) Representation of the activation barriers from the linear transit scans of the multiple conformations extracted from the MD and the initial model (Min.). (B) Representation of the correlation between activation barriers obtained from the linear transit scan and the sum of the interatomic distances at the R state (*d*_sum_ = *d*(His296 Nε – H1 Ser441) + *d*(Ser441 Oγ – C1 Arg^P1^)).

By analyzing the linear transit scan barriers for the 20 structures (which include the X-ray minimized structure), the lowest and highest barriers were 15.07 and 31.72 kcal mol^–1^, respectively (Figure 4A). Despite the subtle differences observed in the QM region of all structures (in both R and TI states), these account for an energy difference of almost 16 kcal mol^–1^ (Figure S11). Moreover, the structure that presented a higher structural difference in comparison to the others was the structure retrieved from the simulation at 38.52 ns. This structure was the one that led to the highest maximum energy state (31.72 kcal mol^–1^) (Table S3). We stress though that the TSs were not fully characterized.

Nonetheless, these results are very interesting to assess the importance of active site distances for the reaction to occur. When we correlated the sum of the proton transfer and nucleophilic attack distances, *d*_sum_, with the activation energies (Figure 4B), we saw that the structure with the highest barrier (31.72 kcal mol^–1^) had the highest *d*_sum_ value (5.02 Å). Interestingly, this structure was retrieved from a period of the MD simulation (38–45 ns), where there was a significant active site destabilization (Figures S2–S6). If we remove this structure from the analysis, the barriers of the other conformations are comprehended between 15.07 and 24.92 kcal mol^–1^, accounting for a ca. 10 kcal mol^–1^ energy difference.

Furthermore, if we look at conformations retrieved at 94.58 and 94.62 ns, despite being only 4 ps apart, they showed a barrier difference of ca. 5 kcal mol^–1^. This was also the case for the conformations retrieved at 49.66 and 49.76 ns, which were only 10 ps apart. Still, in other closely spaced conformations (e.g., 18.23 and 18.28 ns) these differences were less pronounced.

### Exploring the Contribution of the Oxyanion

We recalculated the Gibbs activation energy without the stabilization effect of the oxyanion hole (details in the Methods section). The barrier was 25.1 kcal mol^–1^ (Table S4). This meant that the oxyanion hole stabilizes the rate-limiting TS by 8 kcal mol^–1^, stressing its role in favoring this reaction mechanism.

Our results were in line with a previous computational study performed on the human fatty acid synthase malonyl-acetyl transferase domain, where the authors observed a decrease in the barrier between 7 and 11 kcal mol^–1^ due to the oxyanion hole stabilization, depending on the substrate.^49^ Our results also agreed with the experimental work of Bobofchak et al.,^50^ where it was concluded that the oxyanion hole contributed 1.5–3.0 kcal mol^–1^ to the stabilization of the TS of trypsin-like serine proteases. We considered that the difference in the value was because we calculated the effect of both hydrogen bonds that participate in the oxyanion hole interactions, whereas Bobofchak et al. measured the effect of a single one by mutating Gly193 (corresponding to our Gly439) with Arg and Pro.

Thus, the inclusion of an electronegative or anionic group capable of interacting with these two residues must be taken into consideration in the drug design process, as it will significantly increase the inhibitor affinity to the enzyme.

### Structural Comparison between TI and the Nafamostat-Derived Phenylguanidino AE Complex

Enzymes catalyze chemical reactions through a preferential stabilization of the TS structure. They bind so tightly the TS that the latter is considered the perfect competitive inhibitor.^51^ In the present case, TS1, TI, and TS2 have very similar free energies, so we will take TI as a reference (refer to TI in Figure 2), as its chemical structure does not have bonds being formed/broken. Several inhibitors have been proposed for TMPRSS2. However, only one of them (nafamostat) has a resolved structure in complex with TMPRSS2 (PDB ID: 7MEQ), resulting in a phenylguanidino covalent complex. Using Open-source PyMOL (version 2.3.0), the X-ray structure of TMPRSS2 after treatment with nafamostat was superimposed to our computationally resolved TI structure. The RMSd of the TMPRSS2 Cα atoms obtained for the two structures was 0.344 Å, which meant that they were structurally very similar. Subsequently, the coordinates of the phenylguanidino moiety of nafamostat were transferred to the TI structure. The interactions between this moiety and the TI were then analyzed.

Concerning the inhibition mechanism, nafamostat has a phenylguanidino moiety that allows the inhibition of trypsin-like proteases by mimicking their substrates (Scheme S1). Moreover, the resemblance between the ester group and a peptide bond allows the reaction to occur with the formation of the TI with a faster reaction rate.^52^ However, contrary to the substrate’s reaction, the phenylguanidino moiety remains covalently bound to the catalytic serine after the first reaction step. The nafamostat-TMPRSS2 covalent complex is resistant to hydrolysis, inhibiting its catalytic activity.^53^

**Figure 5:**
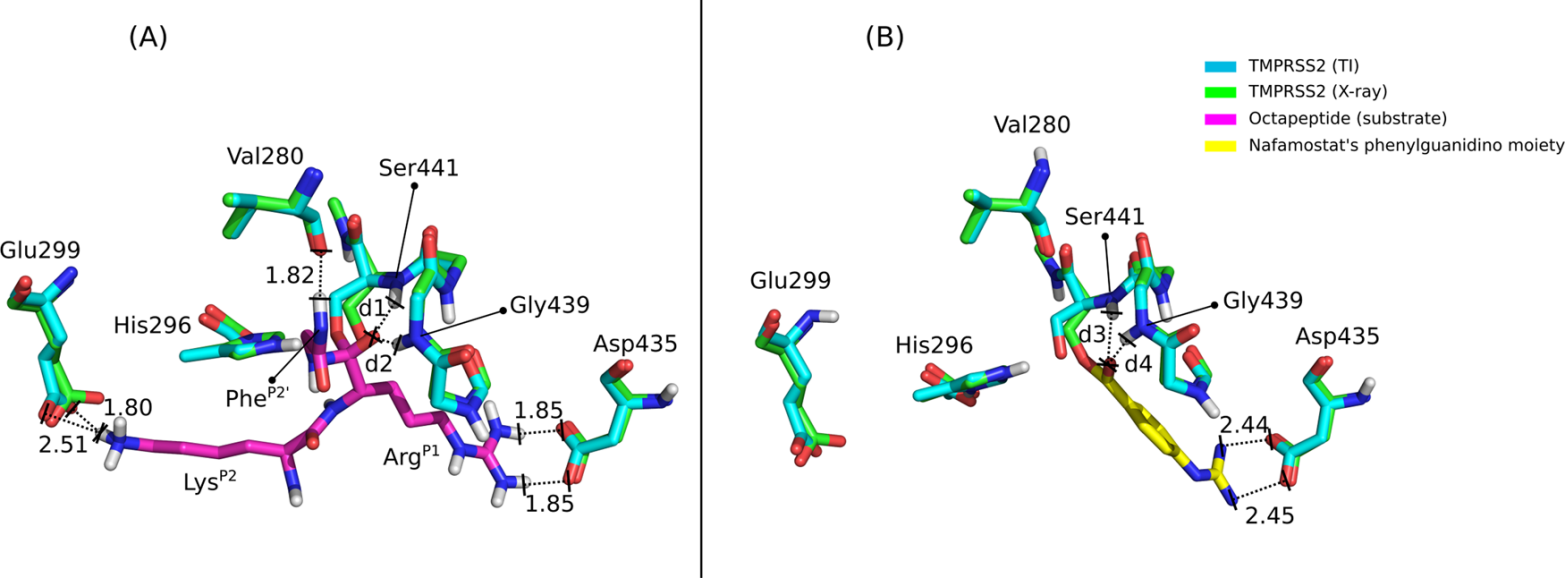
Cross-comparison between the TI structure of TMPRSS2 in complex with the substrate’s octapeptide sequence and the X-ray structure of TMPRSS2 in complex with the phenylguanidino moiety. (A) Structural comparison of the X-ray coordinates of TMPRSS2 and the TMPRSS2:octapeptide complex formed in the TI state. (B) Structural comparison between the TI structure of TMPRSS2 and the TMPRSS2 structure after treatment with nafamostat (X-ray). The formation of the TMPRSS2:Nafamostat AE complex requires that the inhibitor undergoes a proteolytic cleavage. This leads to the formation of a covalent bond between Ser441 Oγ and nafamostat’s phenylguanidino moiety, with the other part of the inhibitor being the leaving group. The TI and X-ray carbon atoms of TMPRSS2 are colored cyan and green, respectively, and the carbon atoms of the substrate and the phenylguanidino moiety are colored magenta and yellow, respectively. The interatomic distances of interacting atoms are represented in Å. The *d*_1_ distance is 1.79 Å, *d*_2_ is 1.74 Å, *d*_3_ is 2.05 Å, and *d*_4_ is 2.58 Å.

In Figure 5, we can see that several polar interactions were established between the enzyme and the inhibitor. The positively charged group of the inhibitor interacted with the carboxylic group of Asp435 (with interatomic distances at 2.44/2.45 Å). The interatomic distance between the participating atoms was higher in the phenylguanidino covalent complex in comparison to the TI structure resolved by our calculations (with an interatomic distance of 1.85 Å), meaning that the interaction strength was higher in the substrate complex.

Unlike the inhibitor complex, the substrate also established a salt bridge between Lys^P2^ and Glu299 (with interatomic distances of 1.80/2.51 Å). Previous molecular docking results showed that nafamostat is not capable of establishing the salt bridge with Glu299.^54^ Nevertheless, keeping these salt bridges might be relevant in the context of inhibitor development. A hydrogen bond interaction was also established between Val280 carbonyl oxygen and Phe^P2′^ amine group (with an interatomic distance of 1.82 Å).

Finally, the calculations show that TI is, in general, similar to the R, but the O1 atom is anionic in TI and neutral at the R. As an enzyme, TMPRSS2 stabilizes more the high-energy TI than the R. This indicates that a negative charge in the O1 region is beneficial for reaching high-affinity inhibitors in future drug design strategies. The negative group at the O1 position should be modeled so that it allows for the formation of hydrogen bonds with the oxyanion hole residues, as these have a stronger binding with the TI state than the R and are the main contributors for the differential stabilization of TI in relation to R in this region.

Increasing the affinity in this manner is particularly relevant for noncovalent inhibitors. In the case of covalent inhibitors, most of the binding free energy is provided by covalent binding. In addition, changing O1 for an anionic species may render C1 less electrodeficient, thus interfering with the barrier and extension of the formation of the covalent adduct.

The phenylguanidino moiety of nafamostat established interactions with both -NH groups that compose the oxyanion hole, with an interatomic distance of 2.05 Å for Ser441 and 2.58 Å for Gly439, a requirement we predict to be fundamental for high affinity. However, the interatomic distances were significantly higher than those established between the enzyme and TI (1.79 and 1.74 Å, respectively), meaning that the oxyanion hole interactions are weaker in the phenylguanidino covalent complex. The reason for these weaker interactions is that nafamostat mimics the neutral O1 of the substrate, but not the anionic O1 of the high-energy TS1-TI-TS2 structures. Nevertheless, the introduction of an anionic group in the O1 position in nafamostat may interfere in the barrier and extension of the covalent binding and not bring an obvious affinity advantage given the already very strong binding of nafamostat.

The introduction of an anionic group at O1 is a promising strategy for the design of noncovalent inhibitors, as it mimics the highest energy states of the free-energy profile, which are the ones the enzyme stabilizes the most. An anionic group is more strongly hydrated, and thus some of the oxyanion hole stabilization will be canceled by the desolvation penalty. However, water molecules need to reorganize to solvate the anionic group, but the enzyme is preorganized to do it from the beginning, with the oxyanion hole hydrogen bonds held in the right place and orientation for the interaction. A vast body of research has shown that the free-energy cost of solvent reorganization, which is almost null at the enzyme preorganized active site (here the cost has been paid already during the folding), forms the basis for the stronger TS binding in enzyme active sites compared to the aqueous solution, and thus the source for enzyme proficiency.^55,56^ Water reorganization has a cost of approximately one-half the hydration free energy. This puts our estimation of the binding free energy gain by introducing an anionic group at the O1 position in ∼4 kcal/mol, which translated into a 2–3 orders of magnitude increase in inhibitor affinity, which is very significant. Even though the hindrances an ionic inhibitor pose to cell membrane passive diffusion decrease the gains made in inhibitor affinity, this is a strategy that is worth trying.

In summary, the structural comparison of the interactions established between the phenylguanidino moiety of nafamostat and TMPRSS2, and the ones established between the substrate and TMPRSS2, revealed important structural features that could contribute to an increase in the affinity between the inhibitor and the enzyme. These features can be helpful in the rational drug development of TMPRSS2 inhibitors.

## Conclusions

The acylation step from the catalytic mechanism of the proteolytic cleavage of SARS-CoV-2 S protein by TMPRSS2 was studied with QM/MM using the ONIOM methodology. The acylation occurred in two sequential steps: (i) a proton transfer from Ser441 to His296 Nε in a concerted manner with a nucleophilic attack of Ser441 Oγ to Arg^P1^ C1 and (ii) a proton transfer from His296 to SerP1′ N1 in a concerted manner with the cleavage of the ArgP1-SerP1′ peptide bond. The oxyanion hole stabilized the rate-limiting TS by 8 kcal/mol. Our results agreed with a literature proposed mechanism for a serine protease associated with the Zika virus.

The first step exhibited a higher Gibbs activation energy than the second, relative to the R state (17.1 vs 15.8 kcal mol^–1^). Furthermore, the formation of the TI was highly endothermic (15.7 kcal mol^–1^), and the energetic difference between the TI state and TS2 state was 0.1 kcal mol^–1^, suggesting that the TI is very short-lived and the transition between these two states happens very quickly. The formation of AE was less endothermic than the formation of the TI (4.5 kcal mol^–1^).

The structural comparison between the phenylguanidino moiety of nafamostat in complex with TMPRSS2 and the TI structure showed similar structures. Nevertheless, the phenylguanidino moiety of nafamostat established hydrogen bonds with the oxyanion hole that were significantly weaker than TI because the former mimics the neutral O1 of the substrate and not the anionic O1 of the TS. In addition, the inhibitor also established a salt bridge with the carboxylic group of Asp435, but weaker than the TI, and lacked a second salt bridge that the TI establishes. All these aspects should be taken into consideration for the design of new, higher-affinity covalent inhibitors and new noncovalent inhibitors.

The thermochemical and structural information provided here can thus be used in future studies to develop resistance-free, efficient drugs for SARS-CoV-2 infection treatment based on inhibitors of the human TMPRSS2 enzyme.

### Data and Software Availability

The force-field parameters and the Cartesian coordinates of the optimized stationary points are included in the Supporting Information. The starting PDB structure (7MEQ) can be downloaded from https://www.rcsb.org/structure/7meq. For the preparation and minimization of the system, AMBER 18 was used. The AMBER package can be purchased on https://ambermd.org/index.php. For the pKa prevision study, DelPhiPKa web server was used ( http://compbio.clemson.edu/pka_webserver/). For visualization of the system, VMD v1.9.4 was used. VMD can be downloaded from https://www.ks.uiuc.edu/Research/vmd/. The VMD molUP extension was used to create the Gaussian input files and to visualize the Gaussian output files. The molUP extension can be downloaded from https://biosim.pt/molup/. For the QM/MM calculations, Gaussian 09 D01 was used. The Gaussian software can be purchased from https://gaussian.com/.

## Supporting Information Available

Additional structural comparisons, energetic data, and atomic charge variation (PDF)
PDB files of the five stationary points, Gaussian inputs for the stationary point optimizations, parameter files for the initial system, and Restart file of the minimized structure (ZIP).
