# Supplementary material for: Transmembrane Protease Serine 2 Proteolytic Cleavage of the SARS-CoV-2 Spike Protein: A Mechanistic Quantum Mechanics/Molecular Mechanics Study to Inspire the Design of New Drugs To Fight the COVID-19 Pandemic
Source: J Chem Inf Model. 2022 May 12;62(10):2510–21. doi: 10.1021/acs.jcim.1c01561 (PMC9113003; doi:10.1021/acs.jcim.1c01561)
Supplement: Supplementary file 1 — ci1c01561_si_001.pdf [file ci1c01561_si_001.pdf]

Supporting Information for

TMPRSS2 Proteolytic Cleavage of the SARS-CoV-2 Spike Protein: A Mechanistic QM/MM Study to Inspire the Design of New Drugs to fight the COVID-19 pandemic.

*Luís M. C. Teixeira, João T. S. Coimbra, Maria João Ramos, Pedro Alexandrino Fernandes **

LAQV/REQUIMTE, Departamento de Química e Bioquímica, Faculdade de Ciências

Universidade do Porto, Rua do Campo Alegre, s/n, 4169-007 Porto, Portugal

* corresponding author: pafernan@fc.up.pt

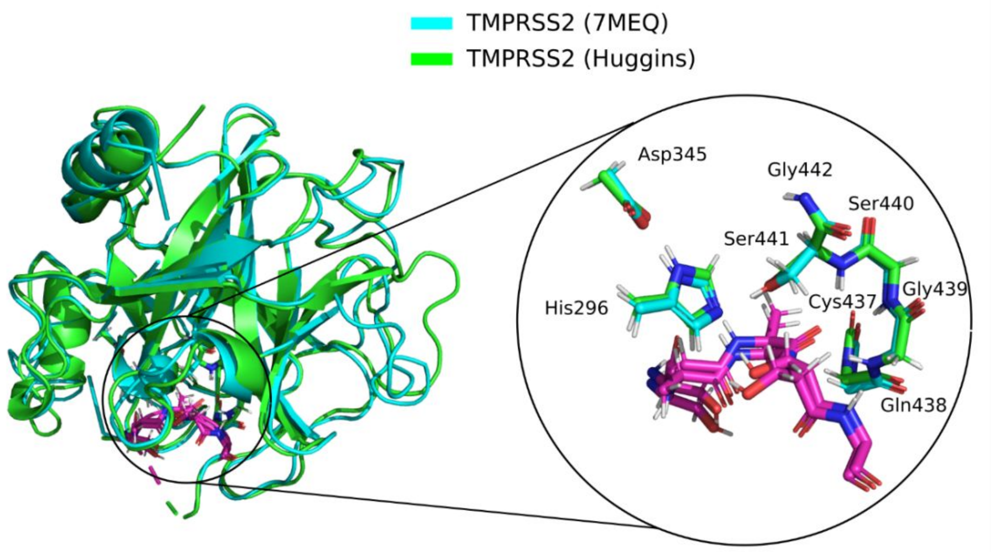

Figure S1 – Structural comparison between TMPRSS2 crystallized structure and the homology model by Huggins [1]. Representation of the secondary structure (left) and the QM region (right) of both models. The carbon atoms and secondary structure of the minimized system are represented in cyan, meanwhile the ones from Huggins's model are represented in green. The carbon atoms from the substrate are represented in magenta.

MOLECULAR DYNAMICS SIMULATION ANALYSIS

In order to characterize the enzyme:substrate complex, we have performed a molecular dynamics (MD) simulation study. This simulation was done after two minimization steps and one water relaxation step, which were performed to obtain a starting enzyme:substrate complex for the QM/MM study. Our MD protocol consisted of four additional steps: 1) system heating; 2) system relaxation; 3) equilibration; and 4) production.

The system heating step was employed during 200 ps, with a starting temperature of 0.0 K and at a constant volume. The temperature was increased every 0.5 ps until the system reached 300 K. Then, the temperature was maintained with a weak-coupling algorithm [2]. During this step, the protein and the octapeptide residues were restrained with a restrain weight of 5.0 kcal/(mol·Å^2) (this was also the case for the following system relaxation step).

The system relaxation step consisted of a 300 ps simulation with a reference temperature of 300 K. A Langevin dynamics protocol with a collision frequency of 1.0 ps^{-1} was used [2]. Concerning the system pressure, an isotropic position scaling with a pressure relaxation time of 2.0 ps and a Berendsen barostat [3] were employed to maintain a 1 bar pressure.

The last two steps did not have any restraints applied. The equilibration step consisted in a simulation of 2 ns, with the same reference temperature and scaling method as the one employed in the system relaxation step, except for the collision frequency, which in this step was 2.0 ps^{-1} (this was also the case for the production step).

The production step consisted in a simulation of 100 ns. In every 5000 integration steps the coordinates were saved. Like the system relaxation step, the pressure of the system was maintained using an isotropic position scaling with a pressure relaxation time of 2.0 ps. However, in this case we employed a Monte Carlo barostat [2].

To evaluate the structure and stability of our system during the simulation, we measured the root mean square deviation (RMSd) of i) all protein atoms, ii) the three catalytic residues atoms, iii) the atoms included in the QM region of the QM/MM study, and iv) all substrate atoms. These measures were done using the minimized structure as a reference (*i.e.*, the starting structure used in the QM/MM study). In addition, the root mean square fluctuation (RMSf) of all substrate's Cα, and the key distances for the reaction were also analyzed.

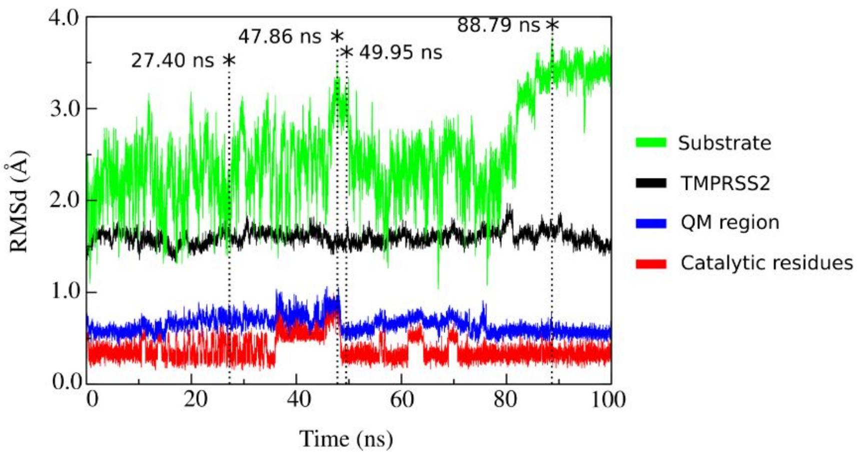

Figure S2 – The root mean square deviation (RMSd) during the production stage. Representation of the four measured RMSd throughout the 100 ns production simulation. All RMSd were measured in reference to the minimized structure used in the QM/MM study. Black, RMSd of all protein atoms; red, RMSd of the three catalytic residues atoms; blue, RMSd of all atoms included in the QM region; and green, RMSd of all substrate residues atoms. Some of the structures were retrieved from the simulation for further visual inspection and are highlighted with "*", along with their corresponding simulation time.

In Figure S2 (black curve), we observed a relatively good stability of the TMPRSS2 protein throughout the simulated time, with an average RMSd of 1.60 Å. Concerning the three catalytic residues (Figure S2, red), they remained stable throughout the entire simulation, having only observed larger fluctuations (but still below 1 Å) in discrete periods of the 100 ns production (*e.g.*

35-48 ns). This was also observed in the atoms that were included in the QM region of the QM/MM study (Figure S2, blue).

In addition, we have also evaluated key distances for the reaction during the production stage. These included the nucleophilic attack and proton transfer distances, the oxyanion distances, and the hydrogen bond distances between the side chains of His296 and Asp345 (Figure S3).

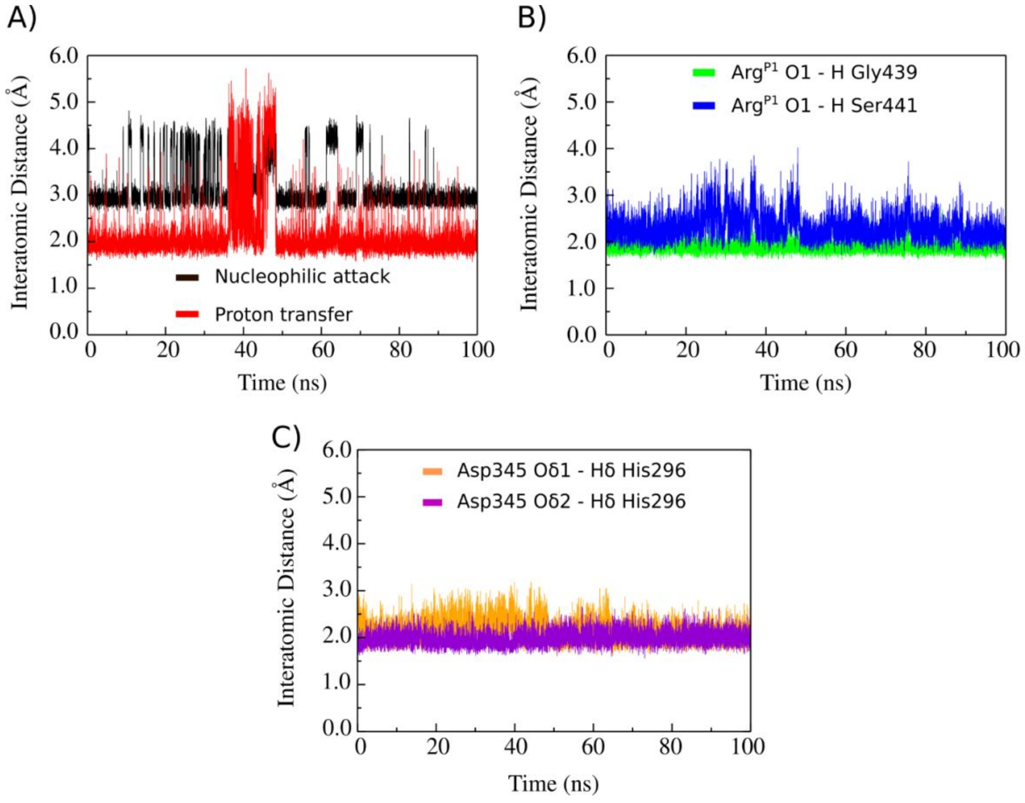

Figure S3 – Variation of key reaction distances throughout the 100 ns molecular dynamics (MD) simulation. A) The distance between Ser441 Oγ and Arg^{P1} C1 (nucleophilic attack) is represented in black, meanwhile the distance between Ser441 H1 and His296 Nε (proton transfer) is

represented in red. B) The distance between Arg^{P1} O1 and Gly H is represented in blue, whilst the distance between Arg^{P1} O1 and Ser441 H is represented in green. C) The distance between Asp345 Oδ1 and His296 Hδ is represented in orange, meanwhile the distance between Asp345 Oδ2 and His296 Hδ is represented in violet.

In Figure S3.A, it is possible to observe that during most of the simulation both interatomic distances remained stable. However, and in agreement with the observation made in Figure S2, between 35 ns and 48 ns there was a higher destabilization of these two distances, with the proton transfer distance showing a larger increase than the nucleophilic attack one.

In Figure S3.B it is possible to observe that the oxyanion hole interactions remained stable during the MD simulation. However, it appears that the interatomic distance between Arg^{P1} O1 and Ser441 H suffers a slight fluctuation throughout the 100 ns simulation.

In Figure S3.C one can observe that the interatomic distance between both Asp345's sidechain oxygen atoms and the His296's Hδ remained stable during the simulation, meaning that the interaction between these catalytic residues was stable. This interaction is important to maintain His296 with the required pK_a for the proton transfer.

Considering both the RMSd analysis and the variation of the key interatomic distances for the reaction mechanism of TMPRSS2, we observed that the active site was stable during most of the simulation. Still, between 35 and 48 ns of the 100 ns production stage, we observed a destabilization of the active site, with pronounced fluctuations in the nucleophilic attack and proton transfer distances (involving the catalytic Ser441).

To understand the reason for the deviation shown on the RMSd graphics and the interatomic distances, five structures from different periods of the simulation, were retrieved. These structures were compared with the minimized structure used in the QM/MM study (Figure S4).

Concerning the structure retrieved at 27.40 ns, in agreement with the observations made during the RMSd and the interatomic distance analysis, it is possible to see that the position of the catalytic residues and the oxyanion hole Gly was very similar. This allowed the enzyme to maintain these residues in the required conformation for initiating the catalytic mechanism. Contrary to this structure, in the structure retrieved at 47.86 ns, we observed that Ser441 suffer a significant change in its orientation. In this structure, the Ser_{cat}'s sidechain rotated, which led to a significant increase between the proton transfer participating atoms. This rotation also led to an increase between the nucleophilic attack participating atoms. Due to this change in orientation, the hydrogen bond

interaction between Ser441's H1 and His296's Nε was broken, resulting in a slightly different conformation of His296. However, the hydrogen bond interactions between His296's Hδ and Asp345 sidechain oxygen atoms were preserved.

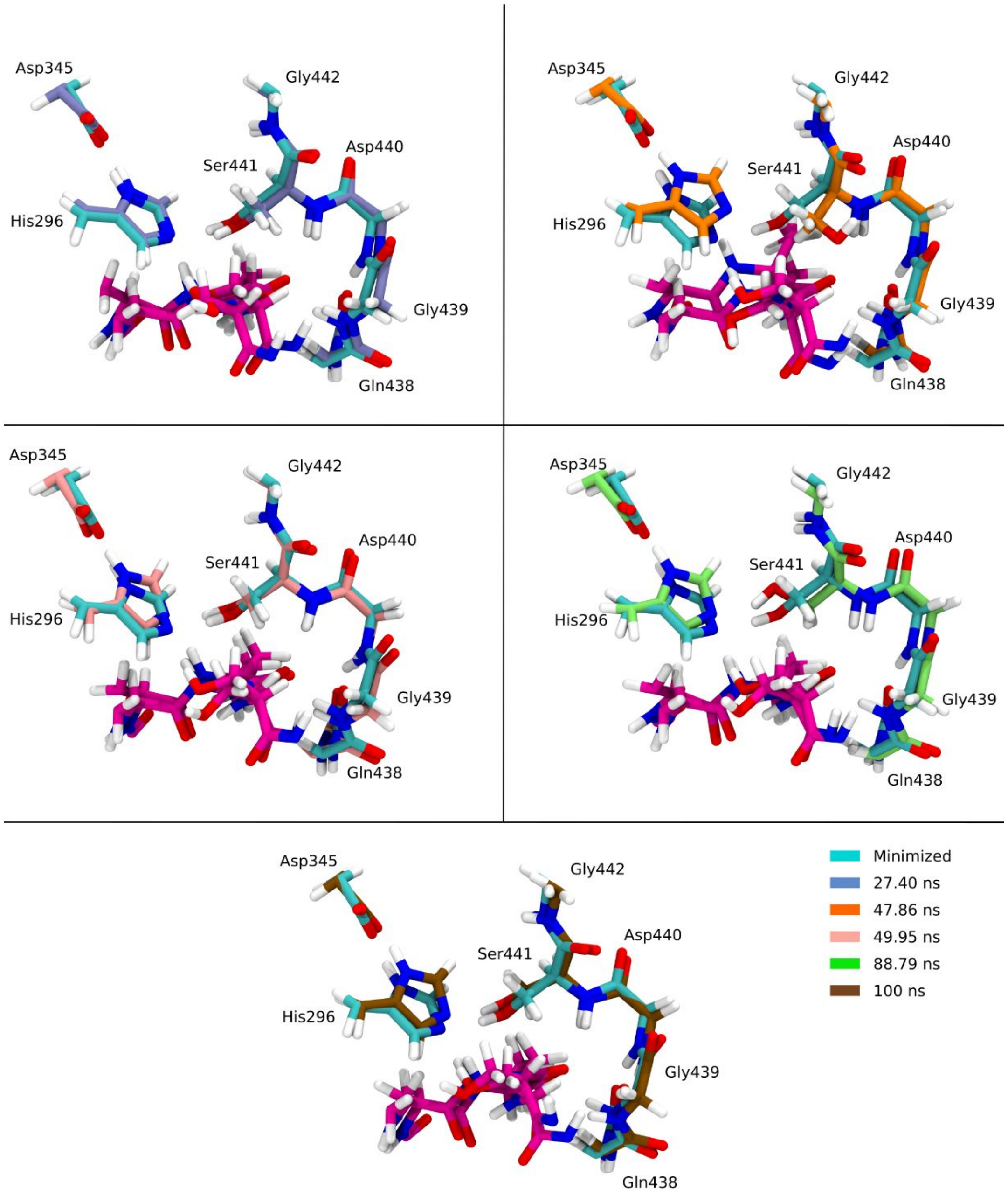

Figure S4 – QM region structural comparison. The minimized structure used as a starting conformation for the QM/MM study was compared with 5 structures retrieved from the 100 ns MD simulation. These structures were retrieved from different periods of the simulation and were

aligned to the minimized structure using VMD. The protein's carbon atoms from the minimized structure are represented in cyan, and different colors were used for representing the different retrieved structures: dark blue, 27.40 ns; orange, 47.86 ns; pink, 49.95 ns; green, 88.79 ns; and brown, the last structure of the MD simulation. In all structures, the substrate's carbon atoms are represented in magenta.

After this period, the graphical analysis showed that the system evolved back to the catalytically competent conformation. In fact, the structure retrieved at 49.95 ns showed that Ser441's sidechain returned to the required orientation, allowing the re-establishment of the hydrogen bond interaction with His296.

Contrary to the protein, the RMSd of all substrate's residues showed that the octapeptide was more flexible, especially in the last 15 ns of the simulation (Figure S3, green). To explore the influence of this flexibility on the QM region, two structures were retrieved: i) one at 88.79 ns, and ii) another at the end of the simulation. Concerning the 88.79 ns structure, we observed that the position of most of the atoms included in the QM region was very similar to the minimized structure, with the exception being Ser441's Oγ. However, the last structure of the simulation showed that this atom evolved back to the required orientation for the reaction. In fact, if we

analyze the nucleophilic and proton transfer distances in the last 15 ns of the simulation, we do not observe a high destabilization of these two distances. This means that the large fluctuation of the substrate probably occurred beyond the P1-P1' region. To explore this effect, we analyzed the RMSf of the substrate's Cα (Figure S5).

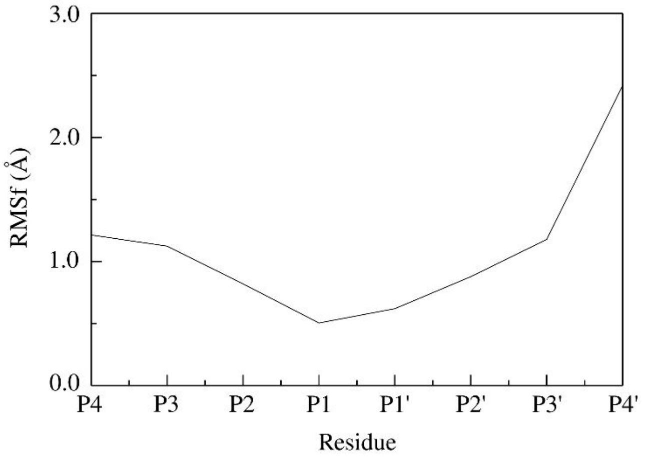

Figure S5 – Substrate's root mean square fluctuation (RMSf). The RMSf was calculated considering only the substrate's Cα.

In Figure S5, one can observe that the residues near the N-terminal and C-terminal of the peptide (and especially the C-terminal), are significantly more flexible than the residues near the middle of the octapeptide. In fact, Arg^{P1} is the most rigid residue in the substrate, with next being $Ser^{P1'}$. So, the high RMSd variation for the ligand during the 100 ns simulations, does not originate in residues of the substrate that are included in the QM region. To confirm this observation, several

structures of the substrate were retrieved from the simulation, and subsequently aligned to compare the conformation of all octapeptide's residues (Figure S6).

The structural comparison of the evolution of the substrate throughout the simulation confirmed the observation made with the RMSf analysis. In fact, one can observe that the residues near both the N-terminal and C-terminal regions are more flexible throughout the simulation, than the ones involved in the reaction. Both, Arg^{P1} and $Ser^{P1'}$ are significantly more rigid, and consequently more stable than Pro^{P4} and $Glu^{P4'}$.

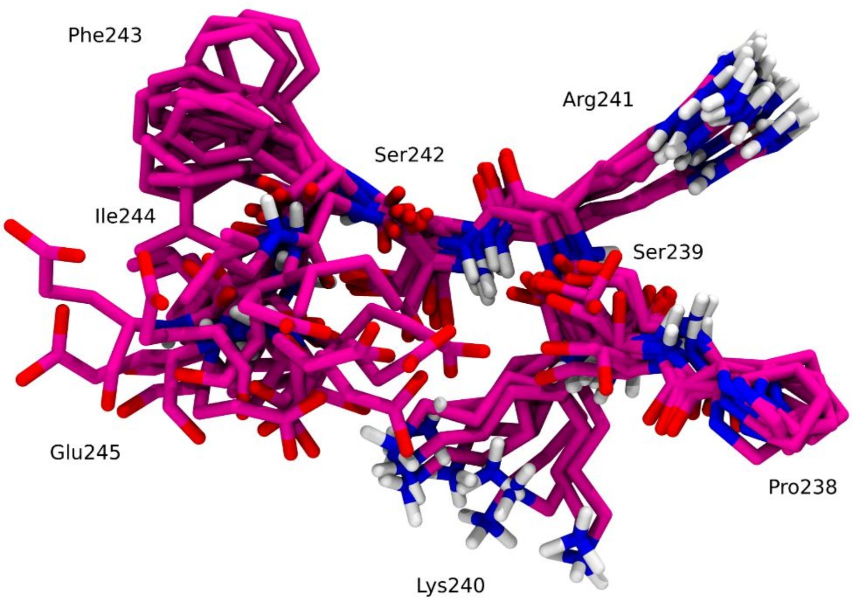

Figure S6 – Substrate conformations along the 100 ns MD simulation. The coordinates of the peptide were retrieved every 10 ns, making a total of 10 conformations. The 10 conformations were then aligned using VMD. For simplicity and visual aid, the non-polar hydrogens were not represented.

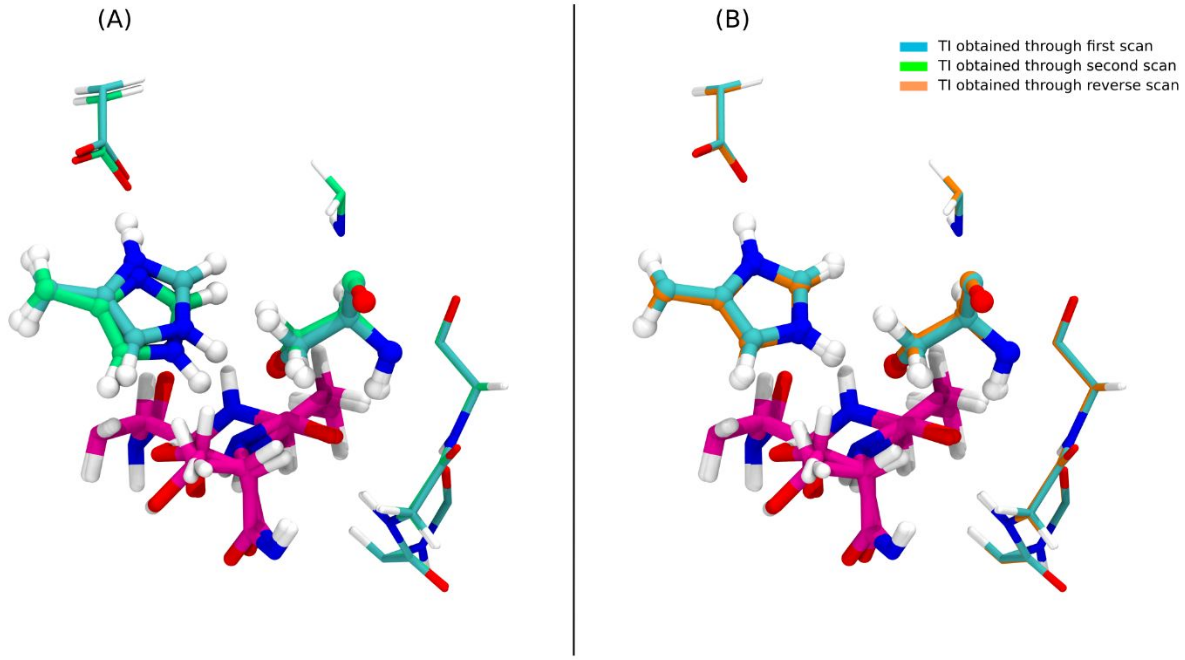

Figure S7 – Tetrahedral Intermediate (TI) structural comparisons. (A) Structural comparison between the TI state obtained through the IRC of the first reactional step, and the one obtained through the IRC of the proceeding step. (B) Structural comparison between the TI state obtained through the IRC of the first reactional step, and the one obtained through the reverse scan protocol. The enzyme's carbon atoms are colored cyan (first scan), green (second scan), or orange (reverse scan). For all structures, the substrate's carbon atoms are colored magenta.

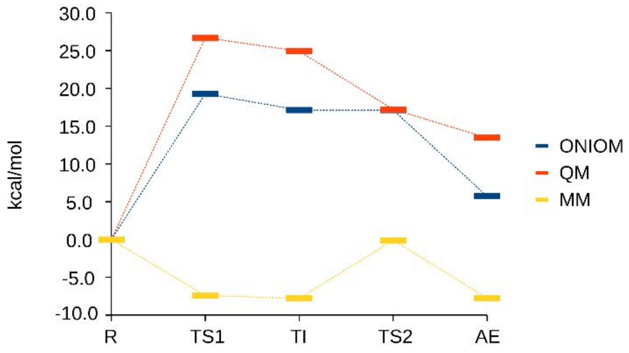

Figure S8 – Graphical representation of the acylation step energies. We show the energy for each stationary point and for each of its three components: i) ONIOM energy (in blue), ii) QM energy (in red), and iii) MM energy (in yellow). All energies are expressed in kcal·mol^{-1}.

Table S1 - Acylation step absolute energies. We show the energy for each stationary point and for each of its three components: i) ONIOM energy (system's energy), ii) QM energy (high layer energy), and iii) MM energy (low layer energy). All energies are expressed in Hartree. The thermal correction to Gibbs Free Energy is also shown.

	ONIOM energy (Hartree)	QM energy (Hartree)	MM energy (Hartree)	Thermal Correction (Hartree)
R	-2697.436595	-2676.498301	-20.93829326	30.452580
TS1	-2697.405848	-2676.455738	-20.95011057	30.449095
TI	-2697.409295	-2676.458583	-20.95071138	30.450250
TS2	-2697.409295	-2676.470842	-20.93845359	30.450428
AE	-2697.427463	-2676.476841	-20.95062226	30.450563

Table S2 – Gibbs Free Energy variation along the acylation step using different levels of theory. The 6-311++G(2d,2p) basis set was used with B3LYP, B3LYP(D3), M06-2X, M06-2X(D3) and BH&HLYP. For comparison, we also show the Gibbs free energy results using the B3LYP/6-31G(d) level of theory (used in the geometry optimizations). All energies are expressed in kcal·mol^{-1}.

	Gibbs Free Energy (kcal·mol^{-1})					
	6-31G(d)	6-311++G(2d,2p)				
	B3LYP	B3LYP	B3LYP-D3	M06-2X	M06-2X-D3	BH&HLYP
R	0.0	0.0	0.0	0.0	0.0	0.0
TS1	16.4	19.6	17.4	17.1	17.0	22.9
TI	16.5	20.8	18.1	15.7	15.5	22.3
TS2	18.1	24.5	18.8	15.8	15.6	26.8
AE	1.0	2.5	4.1	4.5	4.6	3.0

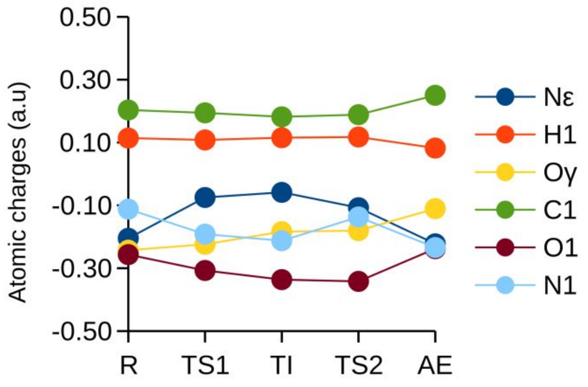

Figure S9 - Atomic charges for the acylation step stationary points. Representation of the Hirshfeld charge variation in atomic units for the main atoms involved in the acylation step. In this graphical representation the connecting lines are only present for visual guidance of the tendency.

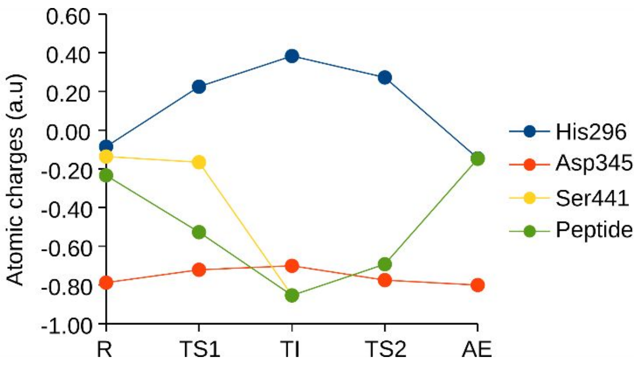

Figure S10 - Acylation step atomic charge variation of the catalytic residues and the substrate. Graphical representation of the variation of the Hirshfeld atomic charge of the catalytic residues (His296, Asp345, and Ser441) and the substrate (peptide) throughout the acylation step. In this graphical representation the connecting lines are only present for visual guidance of the tendency.

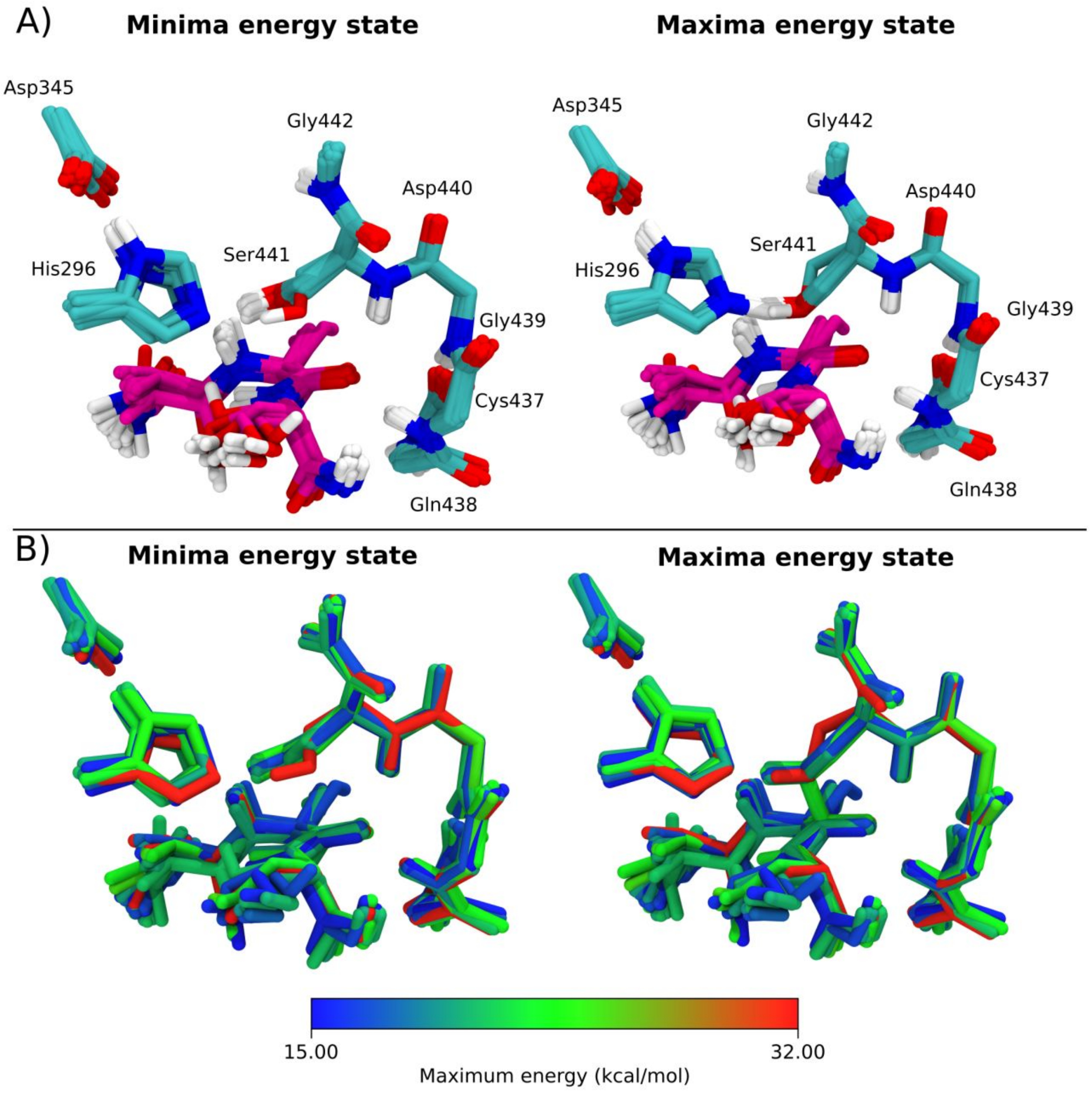

Figure S11 – Representation of the 20 QM regions of the structures corresponding to the energy maxima and minima of each one of the linear transit scans. A) Structural comparison. B) Energetic comparison.

Table S3 – Activation barriers coming from the scan (ΔE^{ONIOM}, calculated at the B3LYP/6-31G(d):FF14SB level of theory) and active site distances after the second QM/MM optimization.

All distances are represented in Å.

Structure	ΔE (kcal/mol)	Proton Transfer	Nucleophilic Attack	Arg^{P1}-Gly439	Arg^{P1}-Ser441	Asp345-His296
Minimized	21.05	1.87	2.76	1.79	1.94	1.61
293	15.96	1.68	2.57	1.80	2.15	1.69
431	24.91	1.71	2.60	1.80	2.08	1.67
841	23.85	1.76	2.74	1.81	2.04	1.80
1823	19.41	1.84	2.67	1.85	2.06	1.63
1828	17.80	1.72	2.64	1.84	2.27	1.78
1918	16.99	1.73	2.66	1.80	2.17	1.64
2906	15.59	1.72	2.62	1.85	2.19	1.76
2918	16.82	1.72	2.60	1.84	1.91	1.73
3207	20.29	1.69	2.64	1.81	2.04	1.68
3852	31.72	1.83	3.19	1.78	2.29	1.59
4966	16.72	1.69	2.58	1.77	2.05	1.68
4976	22.74	1.80	2.66	1.87	2.08	1.73
5814	20.61	1.70	2.62	1.80	2.01	1.67
7090	20.67	1.73	2.66	1.87	2.05	1.76
7202	21.10	1.77	2.65	1.78	2.07	1.79
8030	23.28	1.71	2.63	1.84	2.07	1.65
8612	19.98	1.71	2.61	1.78	2.06	1.66
9458	15.07	1.67	2.59	1.80	2.12	1.73
9462	19.87	1.69	2.64	1.81	2.03	1.68

Table S4 – Energies obtained for the oxyanion hole mutated structures. Representation of the ONIOM energy (in Hartree), the QM energy (in Hartree), and the Gibbs Free energy (in kcal·mol^{-1}) obtained for the R and TS1 structures with the mutated oxyanion hole residues. The thermal correction is also presented in Hartree.

	ONIOM (Hartree)	QM (Hartree)	Thermal Correction (Hartree)	Gibbs Free Energy (kcal·mol^{-1})
R	-2665.357815	-2644.331955	30.45258	0.0
TS1	-2665.314313	-2644.277056	30.449095	25.1

Scheme S1 - The proposed mechanism of inhibition of trypsin-like serine proteases by nafamostat [4]. The first step of the mechanism involves a proton transfer and a nucleophilic attack, which leads to the formation of a tetrahedral intermediate (TI), stabilized through hydrogen bonds with the oxyanion hole residues (a nearby Gly and the backbone of Ser_{cat}). Afterward, a proton is transferred to the leaving group of nafamostat and the ester bond is cleaved, resulting in the formation of an acyl-enzyme (AE).

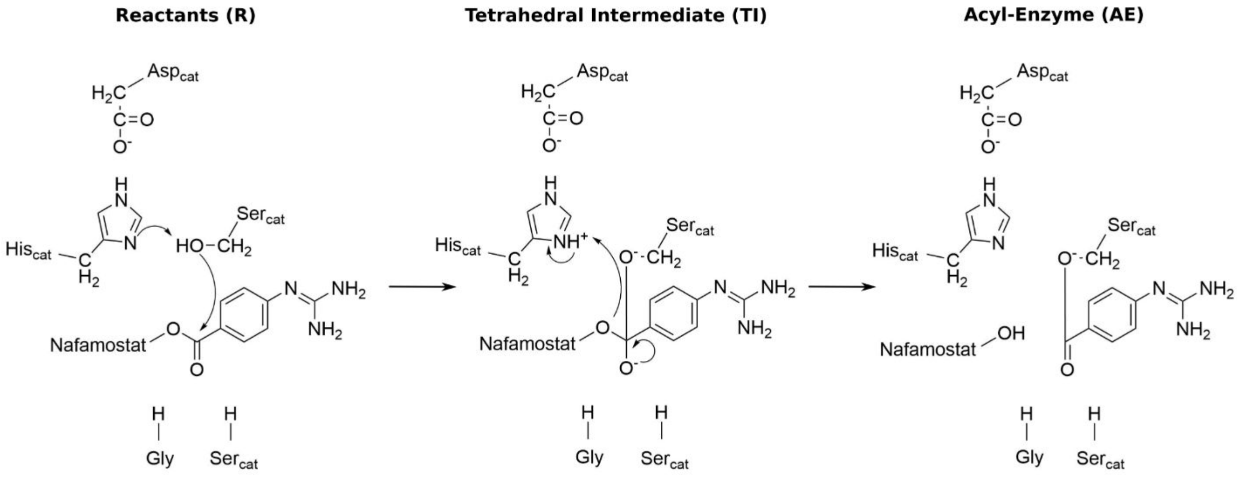

REFERENCE

1. Huggins, D. J., Structural Analysis of Experimental Drugs Binding to the SARS-CoV-2 Target TMPRSS2. *J. Mol. Graphics Modell.* **2020,** *100*, 107710-107710.

2. Hünenberger, P. H., Thermostat Algorithms for Molecular Dynamics Simulations. In *Advanced Computer Simulation: Approaches for Soft Matter Sciences I*, Dr. Holm, C.; Prof. Dr. Kremer, K., Eds. Springer Berlin Heidelberg: Berlin, Heidelberg, 2005; pp 105-149.

3. Berendsen, H. J. C.; Postma, J. P. M.; van Gunsteren, W. F.; DiNola, A.; Haak, J. R., Molecular Dynamics with Coupling to an External Bath. *J. Chem. Phys.* **1984,** *81*, 3684-3690.

4. Hempel, T.; Raich, L.; Olsson, S.; Azouz, N. P.; Klingler, A. M.; Hoffmann, M.; Pöhlmann, S.; Rothenberg, M. E.; Noé, F., Molecular Mechanism of Inhibiting the SARS-CoV-2 Cell Entry Facilitator TMPRSS2 with Camostat and Nafamostat. *Chem. Sci.* **2021,** *12*, 983-992.
